# DDX3X interacts with SIRT7 to promote PD-L1 expression to facilitate PDAC progression

**DOI:** 10.1038/s41389-024-00509-2

**Published:** 2024-02-05

**Authors:** Tianming Zhao, Hanlong Zhu, Tianhui Zou, Si Zhao, Lin Zhou, Muhan Ni, Feng Liu, Hao Zhu, Xiaotan Dou, Jian Di, Bing Xu, Lei Wang, Xiaoping Zou

**Affiliations:** 1 Department of Gastroenterology, Nanjing Drum Tower Hospital, Chinese Academy of Medical Sciences & Peking Union Medical College, Jiangsu Nanjing, 210008 China; 2 Department of Gastroenterology, Nanjing Drum Tower Hospital, Affiliated Hospital of Medical School, Nanjing University, Jiangsu Nanjing, 210008 China; 3 Department of Gastroenterology, Affiliated Taikang Xianlin Drum Tower Hospital, Medical School of Nanjing University, Jiangsu Nanjing, 210023 China; 4 Department of Gastroenterology and Hepatology, Jinling Hospital, Affiliated Hospital of Medical School, Nanjing University, Jiangsu Nanjing, 210002 China; 5 Division of Gastroenterology and Hepatology, Shanghai Institute of Digestive Disease, NHC Key Laboratory of Digestive Diseases, State Key Laboratory for Oncogenes and Related Genes, Renji Hospital, School of Medicine, Shanghai Jiao Tong University, Shanghai, 200127 China

**Keywords:** Pancreatic cancer, Oncogenes

## Abstract

Pancreatic ductal adenocarcinoma (PDAC) is recognized as the most aggressive and fatal malignancy. A previous study reported that PDAC patients who exhibit elevated levels of DDX3X have a poor prognosis and low overall survival rate. However, the underlying molecular mechanism remains unclear. This study aimed to investigate the specific roles of DDX3X in PDAC. Multiple bioinformatics analyses were used to evaluate DDX3X expression and its potential role in PDAC. In vitro and in vivo studies were performed to assess the effects of DDX3X on PDAC cell growth. Furthermore, Western blotting, quantitative PCR, immunohistochemistry, immunofluorescence, mass spectrometry, coimmunoprecipitation and multiplexed immunohistochemical staining were conducted to identify the specific regulatory mechanism in PDAC. The results verified that DDX3X expression is notably upregulated in the tumor tissue vs. normal tissue of PDAC patients. DDX3X knockdown markedly suppressed the proliferation, invasion and migration of PDAC cells in vitro and inhibited tumor growth in vivo. Conversely, overexpression of DDX3X induced the opposite effect. Further studies supported that the DDX3X protein can associate with sirtuin 7 (SIRT7) to stimulate PDAC carcinogenesis and progression. Furthermore, SIRT7 inhibition significantly impeded DDX3X-mediated tumor growth both ex vivo and in vivo. The results also revealed that programmed death ligand 1 (PD-L1) expression is positively correlated with DDX3X expression. These results reveal significant involvement of the DDX3X-SIRT7 axis in the initiation and advancement of PDAC and offer previously undiscovered therapeutic options for PDAC management.

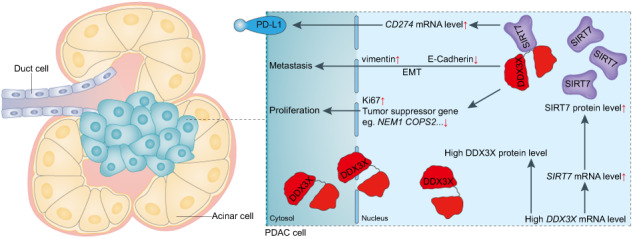

## Introduction

Pancreatic ductal adenocarcinoma (PDAC) is recognized as the most aggressive and fatal malignancy, primarily due to late detection and insensitivity to chemotherapy and ionizing radiation [1]. PDAC exhibits the most dismal prognosis among all major malignancies, with a markedly unfavorable prognosis [2]. In essence, a thorough comprehension of the mechanisms that trigger PDAC and facilitate its progression could pave the way for prompt diagnosis and innovative, targeted treatment.

The X-linked DEAD-box helicase 3 (DDX3X) protein is ubiquitously expressed in organisms. This ATP-dependent RNA helicase takes part in almost all steps of RNA metabolism [3]. Prior studies have indicated that the absence of DDX3X in male mice results in premature lethality in the stage following implantation, highlighting the physiological significance of this protein in regulating development [4]. Given its potential as a target for cancer treatment [5], extensive research attention has been devoted to DDX3X and its corresponding orthologs, resulting in the accumulation of a substantial amount of biochemical and biophysical information. A pioneering study postulated that the DDX3X protein acts as a critical determinant for the survival or death of stressed cells by modulating the activity of the NLRP3 inflammasome [6]. Furthermore, an increasing body of evidence has suggested that dysregulation of DDX3X is commonly observed across different types of cancer [7]. Although the detailed molecular mechanisms are still not fully understood, recent research has demonstrated that high expression of DDX3X is linked to disease advancement and an adverse prognosis in PDAC patients [8]. Conversely, a recent study revealed that DDX3X induces the activation of nuclear factor kappa B (NF-κB) and facilitates metastasis in PDAC through the induction of epithelial-mesenchymal transition (EMT) by regulating p62 [9].

This study demonstrated that PDAC patients with high levels of DDX3X have a poorer prognosis and a lower overall survival rate than those with low levels of DDX3X.

In mammals, the equivalent of Sir2 is known as SIRT, and the SIRT family comprises seven members denoted as SIRT1 through SIRT7; this family regulates various cellular and biological functions that comprise but are not limited to aging, metabolism, genomic stability, and tumorigenesis [10, 11]. SIRT7, a deacylase and ADP ribosylase belonging to the mammalian sirtuin family, is found predominantly in the nucleolus and exhibits widespread expression throughout diverse organs and tissues [12]. SIRT7 is essential for sustaining the malignant characteristics of cancer cells, and its overexpression in diverse cancer types is highly noteworthy [13–15]. Furthermore, O-GlcNAcylation can occur on SIRT7, which subsequently leads to enhancement of pancreatic cancer progression, as this modification regulates it its deacetylation potential and stability [16].

The present investigation sought to examine the potential molecular mechanisms of DDX3X in the progression of PDAC. Notably, our findings indicate that SIRT7 is predominantly downregulated by DDX3X knockdown in PDAC. Therefore, we endeavored to elucidate the underlying regulatory mechanism of SIRT7 by DDX3X in pancreatic cancer. Our findings demonstrate that DDX3X overexpression is crucial for PDAC tumorigenesis and progression. Additionally, we revealed that DDX3X can interact with SIRT7 and that DDX3X overexpression promotes tumorigenesis via a SIRT7-dependent mechanism. Strikingly, we present novel evidence that DDX3X expression correlates with that of the immune checkpoint PD-L1 in PDAC patients and that DDX3X regulates the expression of CD274 (which encodes PD-L1) in PDAC cells. Consequently, the DDX3X-SIRT7 axis holds significant potential for identifying additional targets with valuable therapeutic implications in pancreatic cancer.

## Materials and methods

### Patient specimens

The current study employed PDAC tissue samples and their corresponding peri-tumor tissues that were procured from Nanjing Drum Tower Hospital (Jiangsu, P. R. China) after surgical resection. Before the tissues of any patient were utilized for research purposes, each patient provided written consent. Notably, the study strictly adhered to the rules and regulations of the Institute of Research Ethics Committee at Nanjing Drum Tower Hospital (Jiangsu, P. R. China).

### Cell lines and cell culture

Human PDAC cells (BxPC-3, HPAC, Capan-2, Panc05.04, MIA PaCa-2, PANC-1, SW1990, AsPC-1, and CFPAC-1) were acquired from American Type Culture Collection (ATCC) located in Manassas, VA, USA. Additionally, a standard human pancreatic ductal cell line (HPDE) was procured from Shanghai Cell Bank (Shanghai, China). All cell lines underwent a thorough screening process to detect the presence of mycoplasma, and all were cultivated in DMEM/10% FBS/1.0% Pen-Strep (Wisent, Nanjing, China) at 37 °C.

### Mice

Four-week-old male athymic nude mice (BALB/c nude) were procured from Weitonglihua Biotechnology in Beijing, China. The aforementioned mice were raised in an environment free from pathogens and with strictly regulated temperature conditions, maintaining a 12-hour light-dark cycle at a temperature range of 22–24 °C. Importantly, all animal experiments adhered to the guidelines set forth in the “Guide for the Care and Use of Laboratory Animals” and was authorized by the Institutional Animal Care and Use Committee at Nanjing Drum Tower Hospital.

### Orthotopic Xenograft model

Male athymic mice were administered anesthesia with isoflurane and subsequently injected with either 1 × 10^6^ (PANC-1) or 2 × 10^5^ (SW1990) cells suspended in 50 μL PBS in the tail of the pancreas. Buprenorphine was utilized as an analgesic postoperation. Following tumor growth, measurement of tumor volume was accomplished by employing the formula length × width^2^ × 0.5.

### Subcutaneous Xenograft Model

A total of 1 × 10^6^ cells were injected into the flanks of nude mice at a volume of 100 μL. The tumor volume was assessed through the application of the following formula: length × width^2^ × 0.5. After a period of 2–3 weeks, the mice were subjected to euthanasia, and subsequently, the tumors were dissected.

### Statistical analysis

The data were examined utilizing either the two-tailed Student’s *t* test or one-way ANOVA, followed by post hoc *t* tests. Unless otherwise specified, all data are presented as the mean ± standard deviation. A statistical significance threshold of *p* < 0.05 (*) was adopted for all tests. GraphPad Prism Version 9.0.0 was utilized to conduct the statistical analysis.

The supplementary materials contain information pertaining to additional materials and methodologies.

## Results

### Elevated DDX3X expression is correlated with unfavorable outcomes in PDAC

**Fig. 1:**
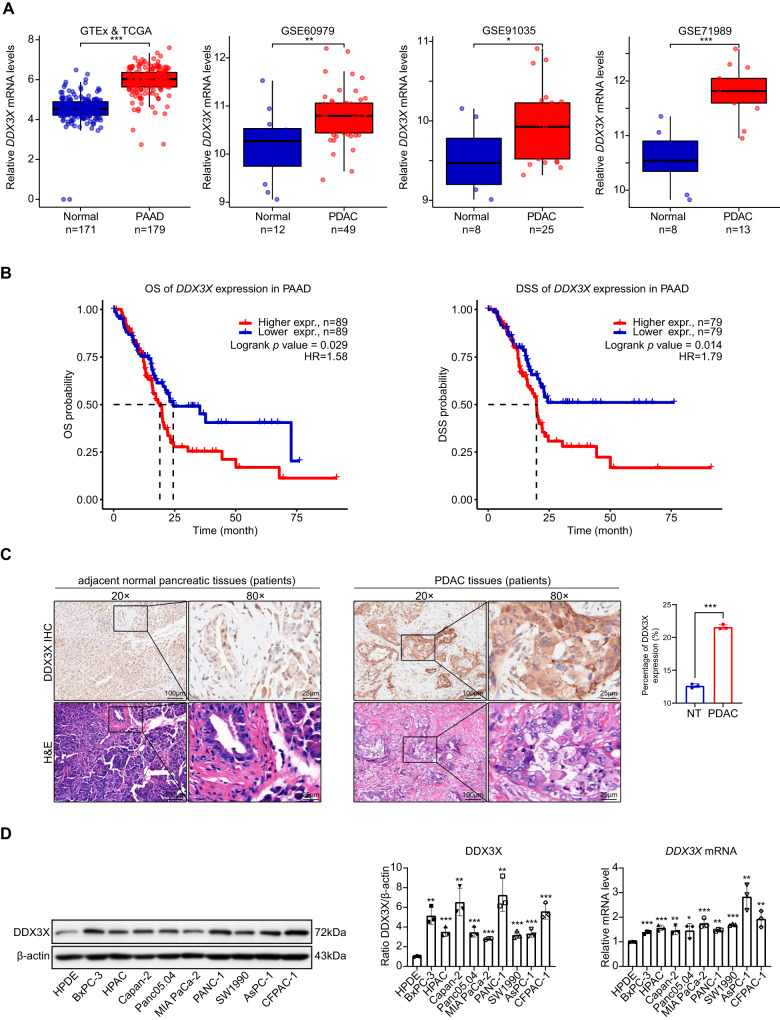
DDX3X overexpression correlates with a poor prognosis in PDAC patients. **A** Gene expression analysis was conducted to compare DDX3X expression between normal pancreatic tissues and PAAD tissues from The Cancer Genome Atlas (TCGA) database, which was combined with the Genotype-Tissue Expression (GTEx) database. To further evaluate the expression of DDX3X between normal pancreas tissues and PDAC tissues, three GSE microarrays from GEO public databases (GSE60979, GSE91035 and GSE71989) were selected for verification. **B** DDX3X expression was assessed to determine its association with overall survival (OS) and disease-specific survival (DSS) in PAAD patients. The median mRNA value was used to categorize tumor samples into high and low-expression groups, and the P values were determined by a log-rank test (*p* = 0.029 and 0.014, respectively). **C** Immunohistochemical staining was performed to detect DDX3X in PDAC/adjacent normal pancreatic tissue samples, accompanied by hematoxylin-eosin (H&E) staining. **D** An analysis of DDX3X protein and mRNA levels was undertaken in human pancreatic ductal HPDE cells and cultured human PC cell lines to further support these findings. Data are shown as the mean ± SD. *p < 0.05, **p < 0.01, ***p < 0.001. *p* values were calculated by Student’s *t* test.

A bioinformatics analysis was undertaken to evaluate the expression levels of DDX3X in various types of cancer to determine its clinical significance in cancer progression. The output from the GEPIA tool demonstrated that DDX3X expression was consistently upregulated in most types of cancer, especially in pancreatic adenocarcinoma (PAAD), when compared to neighboring normal tissues (Fig. 1A and S1A). Moreover, three individual GEO datasets (GSE60979, GSE91035, and GSE71989) confirmed the high expression of DDX3X in PDAC (Fig. 1A). The results of the Kaplan‒Meier survival analysis indicate a notable correlation between elevated DDX3X expression and unfavorable patient survival rates, which is contrary to the outcomes observed in patients with low DDX3X expression (OS *p* = 0.0029; DSS *p* = 0.014) (Fig. 1B). The upregulation of DDX3X at the protein level in tumor tissues was also validated through immunohistochemistry (IHC) staining and H&E staining of human PDAC samples (Fig. 1C and S1B). Furthermore, Western blotting (WB) and IHC were performed on KPC mice compared to WT mice, which confirmed that DDX3X was significantly upregulated in the tumor tissues (Fig. S1C). Additionally, the messenger RNA (mRNA) and protein levels of DDX3X were significantly higher in nine diverse cell lines of human pancreatic cancer than in normal human pancreatic ductal epithelium (HPDE) cells (Fig. 1D). These findings suggest that DDX3X is overexpressed in PDAC tissues and may have an unfavorable effect on patient survival rates.

### DDX3X promotes pancreatic cancer cell proliferation in vitro

**Fig. 2:**
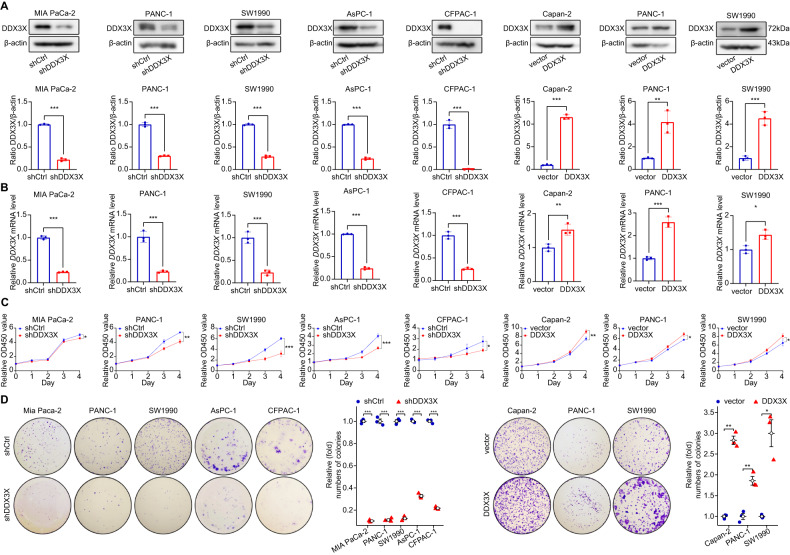
Knockdown of DDX3X suppressed cell proliferation in vitro, whereas overexpression of DDX3X induced the opposite effect. **A**, **B** Validation of DDX3X overexpression and knockdown efficiency was conducted through Western blotting (WB) and RT‒qPCR. **C** The growth curves of PC cells were analyzed using CCK-8 assays. **D** Colony formation assays were performed to evaluate cell proliferation ability. Data are shown as the mean ± SD. *p < 0.05, **p < 0.01, ***p < 0.001. *p* values were calculated by Student’s *t* test.

In light of the identification of DDX3X as an unfavorable prognostic biomarker in PDAC, we undertook an investigation of its specific role in pancreatic cancer. Our approach involved the use of lentiviral short-hairpin RNA (shRNA) to stably silence DDX3X expression in a variety of pancreatic cancer cell lines, namely, MIA PaCa-2, PANC-1, SW1990, AsPC-1, and CFPAC-1. Conversely, we also sought to overexpress DDX3X in Capan-2, PANC-1, and SW1990 cells. Verification of efficient knockdown or overexpression of DDX3X was carried out using Western blotting (WB) and RT-QPCR techniques (Fig. 2A, B). Our CCK-8 assay revealed significant suppression of cell growth upon DDX3X knockdown, whereas overexpression of DDX3X promoted cell growth compared to that in the vector group (Fig. 2C). In addition, we observed that DDX3X depletion led to proliferation inhibition in PC cells, while overexpression of DDX3X stimulated proliferation in Capan-2, PANC-1, and SW1990 cells (Fig. 2D). In summary, our findings suggest that DDX3X promotes cell proliferation in vitro in pancreatic cancer.

### DDX3X promotes migration and invasion through EMT in vitro

**Fig. 3:**
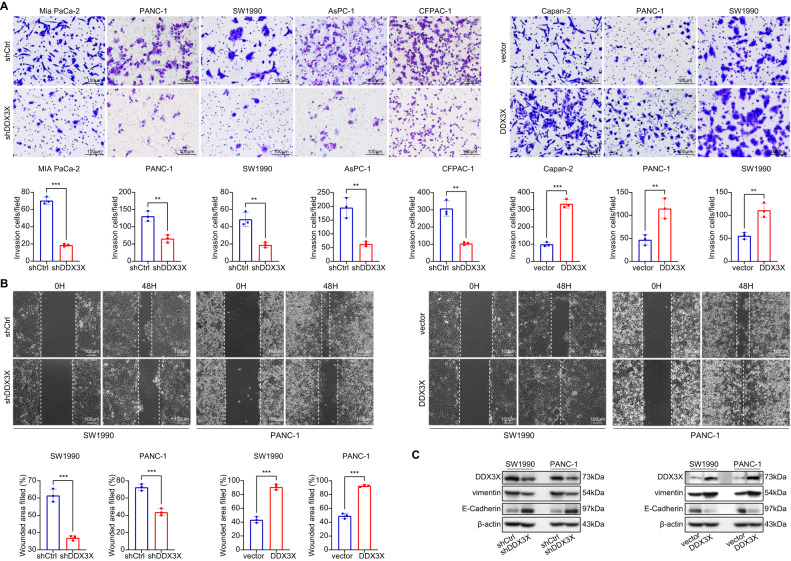
Knockdown of DDX3X significantly inhibited the invasion and migration of PC cells, while overexpression of DDX3X induced the opposite effects. **A** To measure the invasion abilities of PC cells, transwell invasion assays were performed (scale bar = 100 μm). **B** To measure the migration abilities of PC cells, wound healing assays were performed (scale bar = 100 μm). **C** The vimentin and E-cadherin protein levels in the cells were evaluated using Western blotting. The obtained data are presented as the mean ± SD. *p < 0.05, **p < 0.01, ***p < 0.001. *p* values were calculated by Student’s *t* test.

The results of Transwell invasion assays and wound healing assays indicated that the migration and invasion of PDAC cells are suppressed in cells transfected with shDDX3X vs. those transfected with shCtrl. Similarly, upregulation of DDX3X increased the invasion and migration ability of PDAC cells (Fig. 3A, B). To investigate its effects on epithelial-to-mesenchymal transition (EMT) in PDAC cells, Western blot analysis was used and revealed that the silencing of DDX3X increased the expression of E-cadherin and decreased the expression of vimentin in both SW1990 and PANC-1 cells. On the other hand, upregulating DDX3X led to the opposite effect on these EMT markers, as depicted in Fig. 3C. This finding implies that DDX3X could promote the invasion and migration of PC cells through EMT in vitro. Overall, our findings suggest that DDX3X promotes migration and invasion through EMT in vitro and that the expression of DDX3X is closely linked to vimentin-mediated progression in pancreatic cancer.

### DDX3X accelerates tumor growth in vivo

**Fig. 4:**
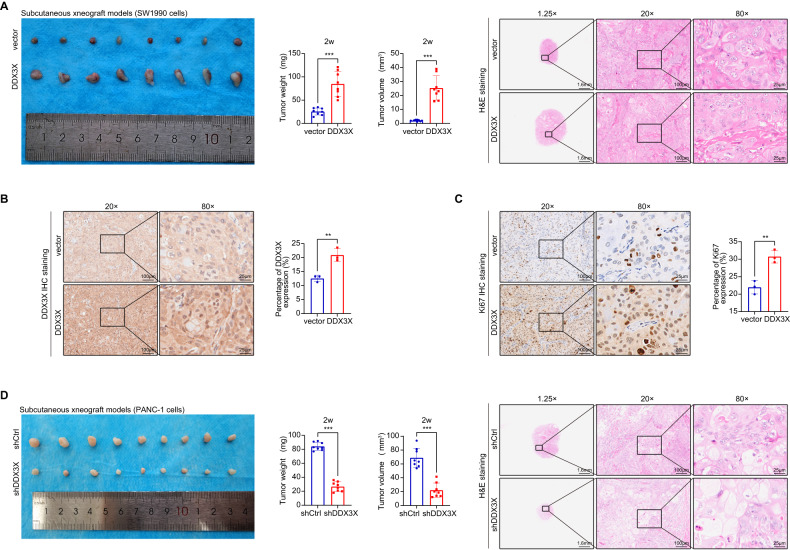
Overexpression of DDX3X promoted proliferation in subcutaneous xenograft models, while knockdown of DDX3X induced the opposite effects. **A** The study, which included eight mice per group, analyzed the weight, volume, and representative images of H&E staining of subcutaneous xenograft tumors. **B** Representative tumor sections stained for DDX3X (IHC). **C** Representative tumor sections stained for Ki67 (IHC). **D** Tumor weight, tumor volume and the images of H&E staining of the tumors (*n* = nine mice per group). Data are shown as the mean ± SD. *p < 0.05, **p < 0.01, ***p < 0.001. *p* values were calculated by Student’s *t* test.

**Fig. 5:**
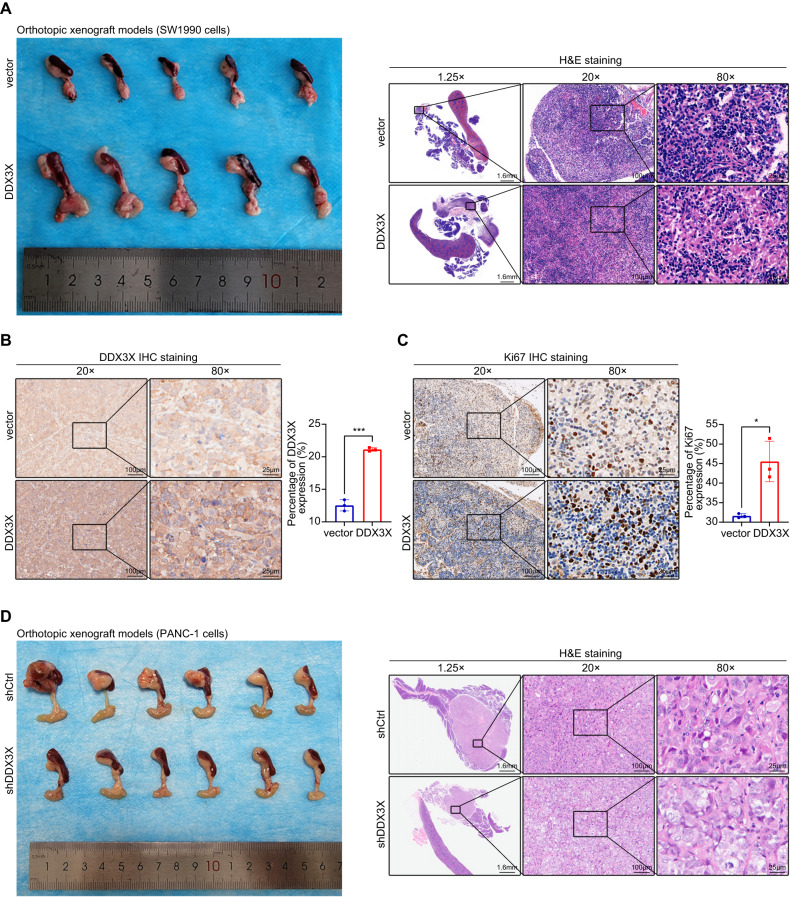
Overexpression of DDX3X promoted proliferation in orthotopic xenograft models, and knockdown of DDX3X induced the opposite effects. **A** Representative images of H&E staining of orthotopic xenograft tumors (*n* = five mice per group). **B** Representative tumor sections stained for DDX3X (IHC). **C** Representative tumor sections stained for Ki67 (IHC). **D** Representative images of H&E staining of the tumors (*n* = six mice per group). Data are shown as the mean ± SD. *p < 0.05, **p < 0.01, ***p < 0.001. *p* values were calculated by Student’s *t* test.

As the in vitro data provided evidence of an oncogenic function of DDX3X in PC cells, we proceeded to investigate its role in malignant tumorigenesis in vivo. Consequently, we infected SW1990 cells with either p-NC or p-DDX3X to establish control or DDX3X-overexpressing pancreatic cancer stable cell lines (vector group and DDX3X group), respectively. Subsequently, the aforementioned cells were administered via subcutaneous injection into the right flank of immunocompromised mice to establish a subcutaneous xenograft model. To validate our findings, we utilized an orthotopic pancreatic cancer mouse model wherein SW1990 cells infected with p-NC or p-DDX3X were implanted into the tail of the pancreas of athymic nude mice. The results demonstrated that DDX3X overexpression could promote pancreatic cancer growth in vivo (Figs. 4A and 5A). Furthermore, to validate the DDX3X expression level in the DDX3X group, we conducted DDX3X immunohistochemistry assays on xenografts and orthotopic tumors. The results revealed that the expression of DDX3X was significantly higher in the DDX3X group than in the vector group (Figs. 4B and 5B). Subsequently, immunohistochemistry (IHC) analysis was performed on xenografts and orthotopic tumors to evaluate the expression of Ki67, which is a commonly employed indicator for assessing cell proliferation. Our observations indicated that, compared to the vector group, the overexpression of DDX3X resulted in an increase in Ki67 staining (Figs. 4C and 5C). To validate our findings and eliminate the potential influence of cell line-specific effects, PANC-1 cells induced with either control or DDX3X-specific shRNAs were subcutaneously injected into the right flank of nude mice for the purpose of conducting the xenograft assay. Next, we generated a pancreatic orthotopic xenograft model. The DDX3X-knockdown xenograft tumors and orthotopic tumors demonstrated substantially reduced growth rates in comparison to the shCtrl tumors (Figs. 4D and 5D). At the endpoint, it was visually clear that the DDX3X-knockdown tumors were smaller than the controls. Taken together, our data indicate that DDX3X promotes tumor growth in vivo.

### DDX3X interacts with SIRT7 in PDAC

**Fig. 6:**
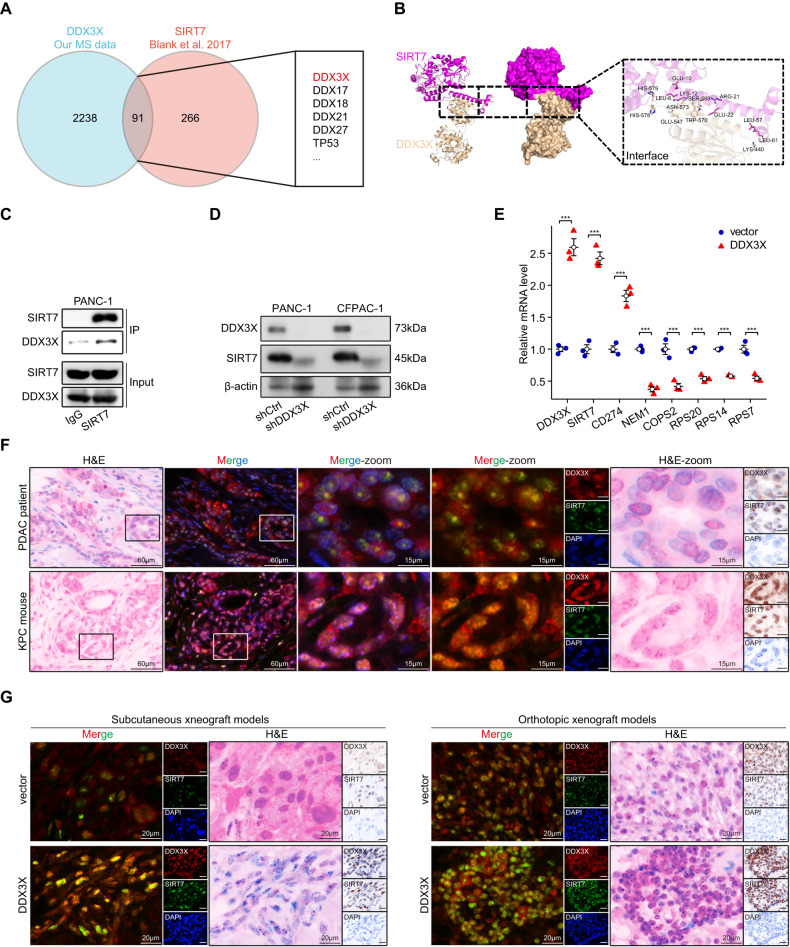
DDX3X binds to SIRT7. **A** Intersection between our mass spectrometry-identified set of DDX3X binding proteins and reported mass spectrometry-identified sets of SIRT7 binding proteins. **B** The interaction of SIRT7 (purple) with DDX3X (gold) predicted by computational protein‒protein docking. **C** An analysis utilizing coimmunoprecipitation was conducted to evaluate the impact of SIRT7 on the interaction with DDX3X in PANC-1 cells. **D** The protein level of SIRT7 in PC cells was examined using Western blot analysis following knockdown of DDX3X. **E** Expression of SIRT7, CD274, NEM1, COPS2, RPS20, RPS14 and RPS7 was checked in DDX3X-overexpressing PANC-1 cells. **F** Representative fluorescent multiplexed immunohistochemical staining (MIF) sections of DDX3X (red) and SIRT7 (green) expression and distribution in PDAC patient tissues and KPC mouse tissues. **G** Representative fluorescent multiplexed immunohistochemical staining (MIF) sections of DDX3X (red) and SIRT7 (green) expression and distribution in subcutaneous xenograft tumors and orthotopic xenograft tumors.

To identify potential interaction partners with DDX3X, mass spectrometry analysis was conducted on HEK293T whole-cell lysate that was purified using tandem affinity. A total of 2,334 proteins were identified. However, given our specific interest in the sirtuin family of proteins, we focused on SIRT7, who has been recently reported to bind DDX3X. Notably, the application of mass spectrometry in the evaluation of proteins linked to human SIRT7 has revealed an extensive presence of DEAD/H box RNA helicases within the network of interactions associated with SIRT7 (Blank et al. [17]; Lee et al. [18]). Furthermore, DDX3X was identified as a candidate in both studies (Fig. 6A). Our analysis utilizing the GEPIA tool (Fig. S2A) revealed that the expression of SIRT7 was upregulated in pancreatic adenocarcinoma (PAAD) tissue compared with adjacent normal tissue. Furthermore, high expression of SIRT7 in PDAC was confirmed in three individual GEO datasets, namely, GSE60979 (*p* = 0.0036), GSE91035 (*p* = 0.0064), and GSE71989 (*p* < 0.0001) (Fig. S2A). Finally, we investigated the association between the mRNA expression levels of SIRT7 and DDX3X by utilizing the LinkedOmics web tool. The results of our study indicate a positive relationship between SIRT7 mRNA and DDX3X mRNA in pancreatic cancer patient specimens (Fig. S2B). This finding is further supported by molecular docking experiments, which suggest that SIRT7 may serve as a target protein for DDX3X (Fig. 6B). Coimmunoprecipitation assays performed on PANC-1 cells provided additional evidence of the interaction between DDX3X and SIRT7 (Fig. 6C). Furthermore, Western blot assays demonstrated a significant decrease in SIRT7 expression following DDX3X knockdown in PC cells (Fig. 6D). Overexpression of DDX3X was found to downregulate tumor suppressor genes in PANC-1 cells (Fig. 6E). To investigate the potential correlation between SIRT7 and DDX3X in PDAC samples, multiplex immunofluorescence staining was utilized to evaluate the expression of both proteins. In vivo analyses of pancreatic tumors from human and murine PDAC samples demonstrated colocalization of DDX3X and SIRT7, suggesting that SIRT7 may be subject to DDX3X regulation (Fig. 6F). Although DDX3X was mainly detected in the cellular membrane and cytoplasm, it was also found in the nucleus. Conversely, SIRT7 was predominantly expressed in the nucleus, as specified in the preceding section. Immunofluorescence staining carried out on xenografts and orthotopic tumors revealed elevated levels of DDX3X and SIRT7 in the DDX3X group compared to the vector group (Fig. 6G). Collectively, these findings suggest the existence of a positive association between SIRT7 and DDX3X in PDAC.

### SIRT7 is needed for DDX3X function

**Fig. 7:**
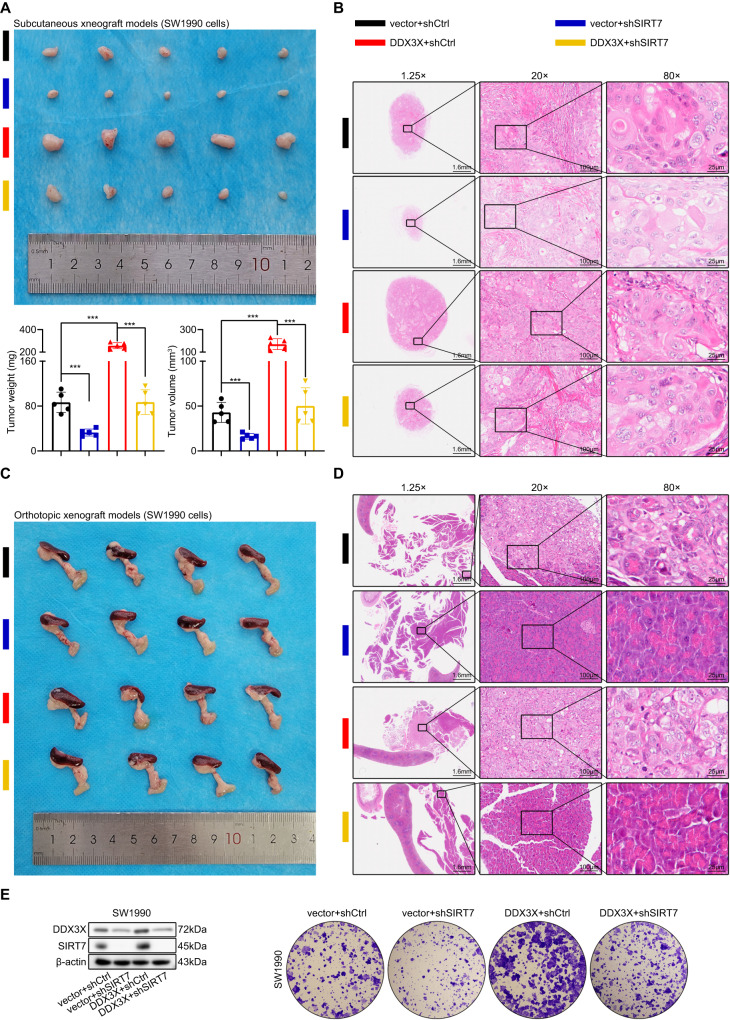
Knocking down SIRT7 reversed DDX3X-induced PDAC proliferation. **A** The weight and volume of subcutaneous xenograft tumors (*n* = five mice per group). **B** Representative images of H&E staining of the tumors. **C** Images of orthotopic xenograft tumors (*n* = four mice per group). **D** Representative images of H&E staining of the tumors. **E** Western blotting was conducted to validate the efficiency of overexpression of DDX3X and knockdown of SIRT7. Cell proliferation ability was evaluated using colony formation assays.

The precise mechanisms through which DDX3X regulates SIRT7 expression in human pancreatic ductal adenocarcinoma (PDAC) cells have not been previously reported. Our investigation revealed that the protein expression level of SIRT7 in human PDAC cells is decreased upon DDX3X knockdown. To determine whether DDX3X contributes to PDAC development in a SIRT7-dependent fashion, we evaluated the impact of DDX3X overexpression-induced SIRT7 suppression on PDAC proliferation. Although DDX3X overexpression significantly promoted PDAC proliferation, the tumor-promoting effects were partly counteracted by SIRT7 suppression in the subcutaneous xenograft model. Our results for tumor weight and volume suggest that stable DDX3X overexpression promotes tumor growth compared to that in the control group, while SIRT7 knockdown suppressed the tumor growth-promoting effects of DDX3X overexpression on tumor formation (Fig. 7A, B). In addition, we verified this phenomenon in a pancreatic orthotopic xenograft model (Fig. 7C, D). We also investigated whether SIRT7 regulates DDX3X in vitro and observed that SIRT7 knockdown partly attenuated the increase in colony formation ability induced by DDX3X overexpression (Fig. 7E), suggesting that the pro-oncogenic influence of DDX3X occurs at least partly through SIRT7. In summary, we conclude that DDX3X overexpression boosts tumorigenesis in a SIRT7-dependent manner.

### Correlation between Ki67/vimentin/PD-L1 and DDX3X in PDAC patients

**Fig. 8:**
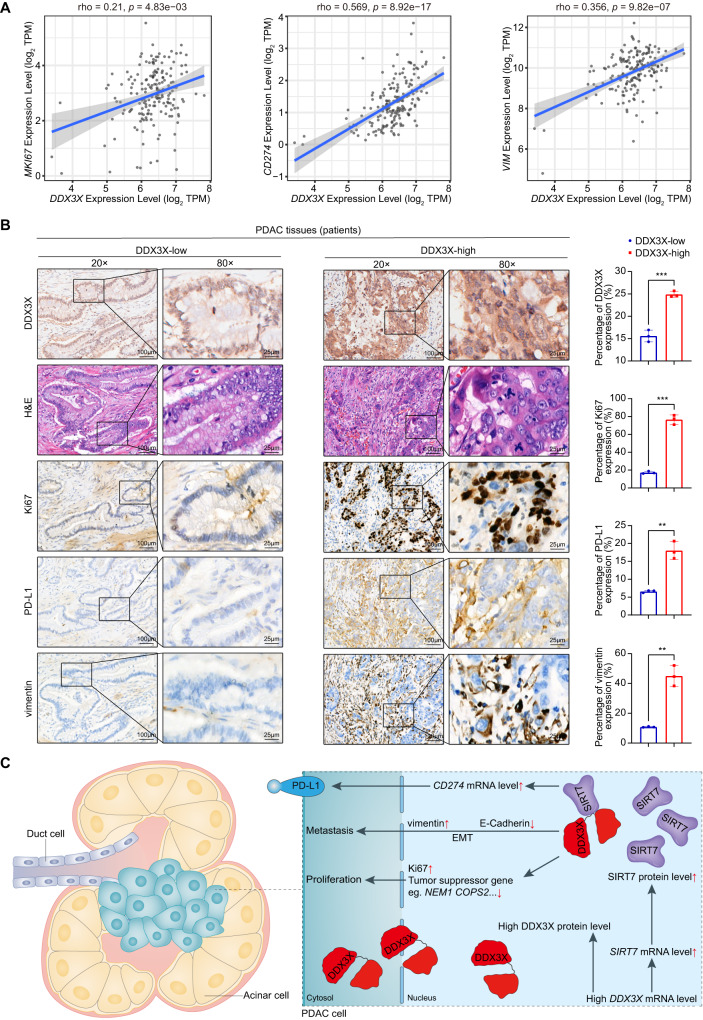
Correlation between DDX3X and Ki67, PD-L1 and vimentin in PDAC patients. **A** Pearson correlation plots of the expression levels of DDX3X and MKI67 (left), CD274 (center), or VIM (right) in the TCGA PAAD cancer dataset. **B** Immunohistochemistry was employed to detect the expression of Ki67, PD-L1, and vimentin in consecutive sections from both the DDX3X-low and DDX3X-high cohorts. **C** A schematic model of how DDX3X and SIRT7 exert functions in PDAC cells.

Considering that DDX3X has been shown to promote tumor proliferation in nude mice, we further investigated whether there was any relationship between the expression patterns of DDX3X and the proliferation marker Ki67 in PDAC patients. Our results demonstrated a positive correlation between Ki67 expression and the DDX3X protein level, which is consistent with the correlation of their mRNA levels reported earlier (Fig. 8A, B). Additionally, consecutive sections were stained to compare the expression of both DDX3X and vimentin in PDAC patients. The results showed that groups with high DDX3X expression also tended to have high vimentin expression (Fig. 8B).

Initially, a correlation analysis was undertaken to investigate the association between the messenger RNA (mRNA) levels of CD274, known as PD-L1, and DDX3X utilizing the GEPIA web tool. The outcomes of this analysis indicate that there is a positive correlation between CD274 mRNA and DDX3X mRNA within PAAD patient specimens, as evidenced by a Pearson’s product-moment correlation coefficient of r = 0.569 and a *p* value of 8.92e-17 (Fig. 8A). To further investigate the correlation between PD-L1 and DDX3X in PDAC specimens, immunohistochemistry (IHC) was performed on a cohort of forty PDAC samples to evaluate the expression of these two proteins. Figure 8B presents representative images obtained from this experiment. The outcomes indicated that there was a positive correlation between the level of PD-L1 expression and that of the DDX3X protein in PDAC specimens, which is congruous with the previously mentioned correlation at the mRNA level. Additionally, upregulation of CD274 mRNA was observed in PANC-1 cells with DDX3X overexpression (Fig. 6E). The obtained outcomes present a strong indication of a strong positive correlation between PD-L1 expression and DDX3X, which signifies the potential of DDX3X as a reliable marker for diagnosis and prognosis and treatment response prediction in pancreatic cancer. It is especially valuable as a marker of the response to immune checkpoint blockade therapy.

## Discussion

The gene designated DEAD-box RNA helicase 3X (DDX3X) encodes an RNA helicase protein that assumes a central role in the development of several carcinomas, such as renal cell carcinoma, hepatocellular carcinoma (HCC), prostate cancer, lung cancer, and colorectal cancer [19–24]. Depending on the specific tumor type, DDX3X may function as either an oncogene or a tumor suppressor. Previous scientific inquiries have revealed that DDX3X is extensively expressed in human CRC tissue and that suppressing this gene could increase the in vivo antitumor activity of antiviral nucleosides (AVNs) including AVN A25 [25]. Additionally, low DDX3X levels have been showed to be correlated with inferior overall survival in patients with RCC, and this phenotype is also correlated with tumor size, lymph node metastasis, and distant metastasis [19]. DDX3X has considerable functional value in various cancer types, so identification of genetic targets linked to this gene is the topic of many research efforts.

In the literature, there is a paucity of comprehensive reports that elucidated the role of DDX3X in the tumorigenesis of PDAC. Consequently, further investigations are necessary in this regard. Previous studies have revealed that interventions directed toward the PAF1-PHF5A-DDX3 complex have the potential to decelerate the progression of pancreatic cancer [26]. Additionally, subsequent research has shown that modification of the bioenergetics of PC cells could effectively influence the functionality of TRIM29 through a collaborative effort facilitated by the recruitment of miR-2355-3p and DDX3X to the AK4 transcript [27]. Recently, a noteworthy finding has emerged, underscoring the indispensable regulatory role of STAU2 in pancreatic adenocarcinoma, and DDX3X was identified as its target gene [28]. In light of these discoveries, one hypothesis suggests that the upregulation of DDX3X is a contributing factor to the advancement and dissemination of pancreatic carcinoma. A deeper understanding of DDX3X’s significant role in PDAC can enhance our comprehension of the biological mechanisms underlying its progression and potentially provide an optimal target for therapeutic approaches aimed at PDAC patients.

The investigation revealed a significant correlation between high DDX3X expression in pancreatic ductal adenocarcinoma (PDAC) tissues and cells and an unfavorable prognosis for PDAC patients. Notably, DDX3X upregulation promoted PDAC proliferation in vitro, while DDX3X knockdown inhibited proliferation. The aforementioned outcomes were additionally confirmed in vivo through the utilization of mouse subcutaneous xenograft tumor models and orthotopic xenograft tumor models. Hence, these discoveries suggest that DDX3X plays a pivotal role in the advancement of PDAC. Nevertheless, an in-depth investigation is needed to determine the precise mechanism by which DDX3X exerts its effects.

Previous studies have shown that DDX3X induces epithelial-mesenchymal transition (EMT) through p62/Sequestosome-1 in vitro and in vivo, promoting PDAC metastasis. We presented a simplified question: Does upregulating DDX3X enhance the invasion and migration abilities of PDAC cells while reducing the expression of E-cadherin (an epithelial marker) and enhancing the expression of vimentin (a mesenchymal marker) in SW1990 and PANC-1 cells? Our CCK-8 assay revealed significant suppression of cell growth upon DDX3X knockdown, whereas overexpression of DDX3X promoted cell growth compared to that in the vector group on day 4. But on day 2 (48 h), there were no significant differences between the two groups in cell viability (Fig. 2C). Therefore, we could exclude in Fig. 3 that these effects on migration and invasion are not the result of hampered cell viability in 48 h. Overall, the data suggest that DDX3X induces migration and invasion through EMT in vitro, consistent with reported findings.

It has been suggested that sirtuins, which rely on NAD^+^ for deacylation, play a role in regulating aging and longevity [29]. In mammals, a total of seven sirtuin members oversee various critical biological processes; however, their involvement in carcinogenesis remains a contentious topic [12, 30–32]. Particularly noteworthy is the fact that SIRT7 is the sole sirtuin that exhibits localization in the nucleolus of cells, and its absence in mice leads to genomic instability and accelerated aging [33]. Although SIRT7 is overexpressed in various types of tumors, there is inconsistent evidence in the literature regarding its role in tumor development [34–36].

Previous research has demonstrated that the depletion of SIRT7 considerably inhibits cell proliferation in various types of malignancies, including breast cancer, prostate cancer, and thyroid cancer [15, 37–39]. Moreover, SIRT7 plays a crucial role in promoting liver cancer metastasis [35]. Conversely, a subsequent investigation revealed that the repression of SIRT7 may trigger breast cancer metastasis [34]. At the molecular scale, it has been acknowledged that SIRT7 interacts with and stimulates the deacetylation process of P53 [40]. Furthermore, the deacetylation of SMAD4 through SIRT7 has the potential to function as a blocker for TGF-β, which may be of relevance when targeted therapy is needed for appropriate individuals with cancer [14]. Nonetheless, high expression of tumor suppressor genes such as TP53 and SMAD4 is common in PDAC. This suggests the possibility that SIRT7 potentially plays a crucial role in the initiation of pancreatic cancer [41]. Nonetheless, conclusive evidence that supports the exact function of SIRT7 in PDAC is currently scarce. A recent study reported that SIRT7 can become O-GlcNAcylated and stimulate pancreatic cancer progression [16].

The current investigation has demonstrated that SIRT7 expression is upregulated in pancreatic adenocarcinoma tissues vs. adjacent normal tissues. These findings are in agreement with previous findings. Significant decreases in both the mRNA levels and protein levels of SIRT7 were observed upon DDX3X knockdown. In vitro and in vivo experiments indicated that exogenous overexpression of DDX3X, which promotes PDAC proliferation, was partially inhibited following SIRT7 knockdown, suggesting that DDX3X may promote PDAC proliferation via modulation of SIRT7 expression. It is widely acknowledged that O-GlcNAcylation maintains the stability of the SIRT7 protein, which inhibits the transcription of a few tumor suppressor genes [16]. Upon overexpression of DDX3X, the expression of tumor suppressor genes was downregulated. Therefore, overall, it can be concluded that the DDX3X-SIRT7 axis may play a significant role in PDAC carcinogenesis and progression.

Additionally, a notable association between elevated levels of DDX3X expression and positivity for PD-L1 has been identified. The overexpression of DDX3X resulted in the upregulation of CD274 mRNA expression and consequently increased PD-L1 expression, which is known to facilitate immune evasion by various tumor cells [42]. There is considerable debate on whether PD-L1 expression is more closely linked to PD-L1 blockade therapy in nontumor or tumor cells. Previous studies have highlighted the correlation between SIRT7 and PD-L1. Through the regulation of PD-L1 via MEF2D, disrupted SIRT7 expression led to increased checkpoint inhibitor efficacy in HCC cells. Furthermore, in an allograft tumor model, immune-competent mice engrafted with HCC cells that lacked SIRT7 gene expression had significantly higher PD-L1 levels than those engrafted with control HCC cells [43]. Moreover, the overexpression of SIRT7 had an unfavorable impact on the immune response against tumors, as it induced the expression of PD-L1 via the IRE1α-XBP1 pathway [14]. Our data indicate the potential involvement of the DDX3X-SIRT7 axis in modulating PD-L1 expression, which is a novel finding. Therefore, DDX3X holds promise as a target for PD-L1 blockade therapy.

## Conclusion

Collectively, our findings provide a novel perspective on the distinct function of DDX3X in PDAC. We have effectively elucidated the pivotal role of the DDX3X-SIRT7 axis in PDAC. Our research findings suggest that targeting the constituents of this axis holds potential as a therapeutic strategy in the treatment of PDAC. Moreover, high DDX3X expression was found to be correlated with high PD-L1 expression in PDAC patients. This finding could hold considerable significance in increasing the clinical response rate and effectiveness of PD-1/PD-L1 blockade.

### Supplementary information

Supplementary figure legends

Figure S1

Figure S2

Figure S3

Figure S4

Supplementary Table 1

Details on other materials and methods

## Data Availability

The data and materials utilized and/or analyzed throughout the present investigation may be obtained from the corresponding authors upon reasonable appeal.
