# Supplementary material for: DDX3X interacts with SIRT7 to promote PD-L1 expression to facilitate PDAC progression
Source: Oncogenesis. 2024 Feb 5;13(1):8. doi: 10.1038/s41389-024-00509-2 (PMC10844636; doi:10.1038/s41389-024-00509-2)
Supplement: Supplementary file 1 — Supplementary figure legends [file 41389_2024_509_MOESM1_ESM.docx]

**Fig. S1 DDX3X expression is increased in both PDAC tissues and PCLM tissues.** **(A)** A pan-cancer analysis revealed that DDX3X expression was vastly increased in a multitude of cancers; **(B)** Examination of the Human Protein Atlas (HPA) database revealed high expression levels of DDX3X in pancreatic cancer patients (left). Representative multiplexed immunohistochemical staining (MIF) sections of DDX3X expression and distribution in PDAC patient tissues (right); **(C)** Protein expression of DDX3X in wild-type mice and KPC mice was analyzed and is presented (left). Data is shown as mean ± SD, n = six/ group. **p* < 0.05. *p*-values are calculated by Student’s t-test. Representative multiplexed immunohistochemical staining (MIF) sections of DDX3X expression and distribution in KPC mouse tissues (right). **(D)** Representative IHC stained sections of DDX3X in normal liver tissues and PCLM tissues.

**Fig. S2** **Correlation between DDX3X and SIRT7 in PAAD.** **(A)** The transcriptomic profile of SIRT7 was examined in normal tissues of the pancreas and tissues afflicted with pancreatic adenocarcinoma (PAAD), utilizing data extracted from The Cancer Genome Atlas (TCGA) database and the Genotype-Tissue Expression (GTEx) database. For additional confirmation of SIRT7 expression patterns, three GSE microarrays were selected from publicly available GEO databases (GSE60979, GSE91035, and GSE71989) and subjected to further evaluation; **(B)** Correlation analysis of DDX3X mRNA in PAAD was performed using LinkedOmics (left). Pearson correlation plots of the expression levels of DDX3X and SIRT7 in the TCGA-PAAD cancer dataset (right); **(C)** Representative immunofluorescence staining image of human SW1990 cells and mouse KPC cells, stained with DDX3X (ab235940, Abcam, 1:100), SIRT7 (sc-365344, Santa Cruz, 1:50) antibodies.

**Fig. S3 Correlation between DDX3X and PD-L1 in PAAD. (A)** High DDX3X expression is associated with more immune infiltrate in PAAD (TCGA-PAAD, N=177, R=0.38, *p*=2.0e-7). **(B)** Correlation between DDX3X and 60 immunomodulators (24 immunoinhibitors and 36 immunostimulators). **(C)** Survival analysis according to DDX3X mRNA expression. **(D)** Kaplan-Meier analyses of OS in patients in the TCGA-PAAD cohort according to the combination of DDX3X and CD274. Data were analyzed by log-rank test. HRs with 95% CI were estimated by Cox proportional hazards model. **(E)** Representative fluorescent multiplexed immunohistochemical staining (MIF) image of human PDAC tissues, stained with DDX3X (ab235940, Abcam, 1:1000), SIRT7 (sc-365344, Santa Cruz, 1:100), and PD-L1 (66248-1-Ig, Proteintech, 1:5000) antibodies.

**Fig. S4 Tumor weight of orthotopic xenografts. (A)** Tumor weight of the tumors (as a supplement to Fig. 5A). **(B)** Tumor weight of the tumors (as a supplement to Fig. 5D). **(C)** Tumor weight of the tumors (as a supplement to Fig. 7C).
