# Supplementary figures and images for: DDX3X interacts with SIRT7 to promote PD-L1 expression to facilitate PDAC progression
Source: Oncogenesis. 2024 Feb 5;13(1):8. doi: 10.1038/s41389-024-00509-2 (PMC10844636; doi:10.1038/s41389-024-00509-2)

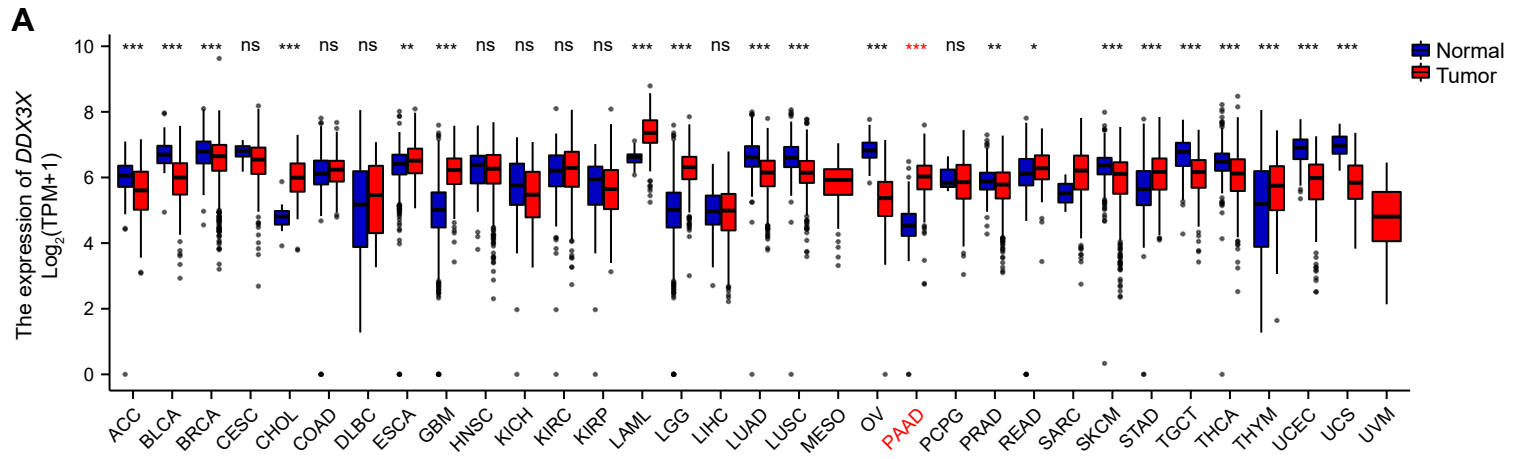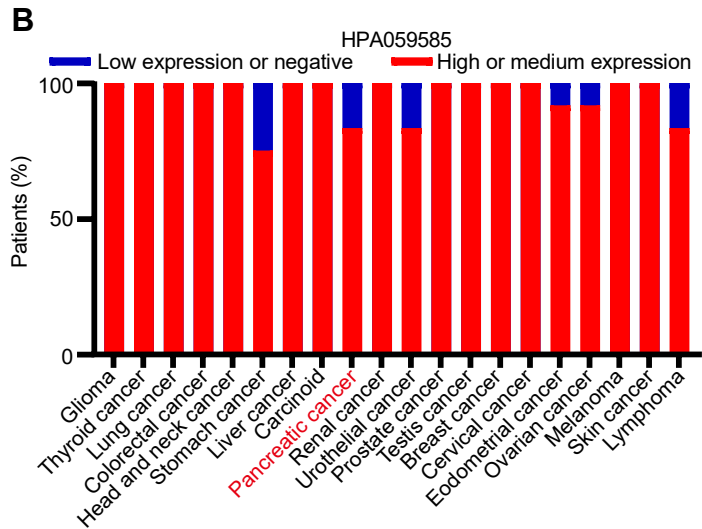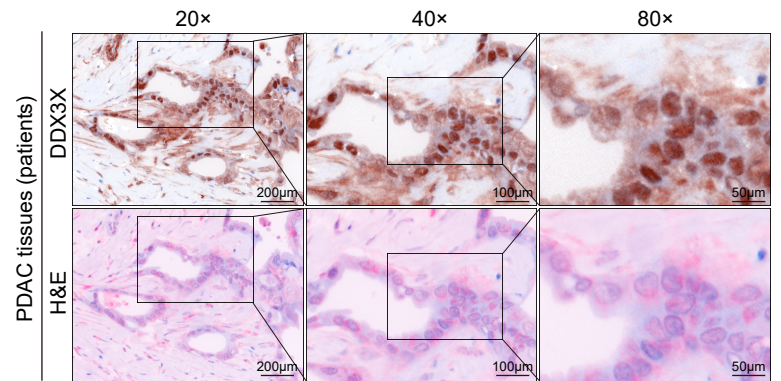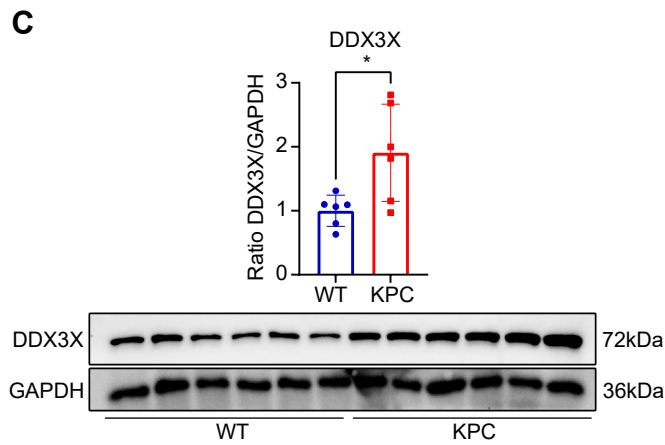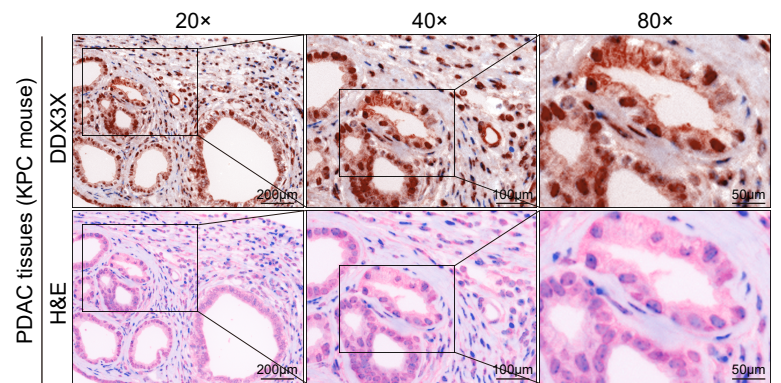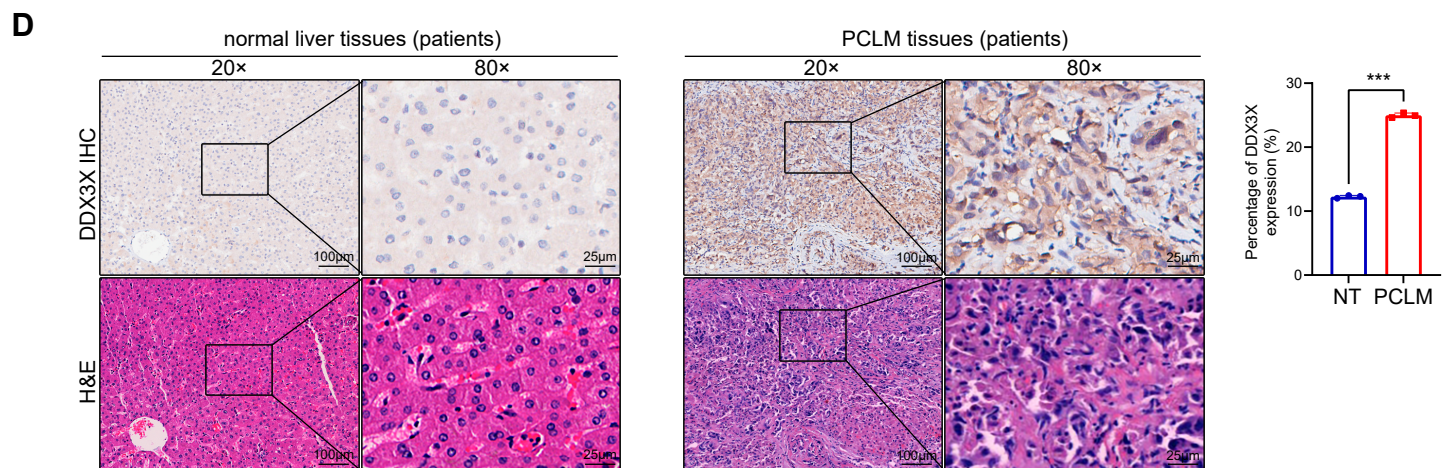

Supplement: Supplementary file 2 — Figure S1 [file 41389_2024_509_MOESM2_ESM.pdf]

**A**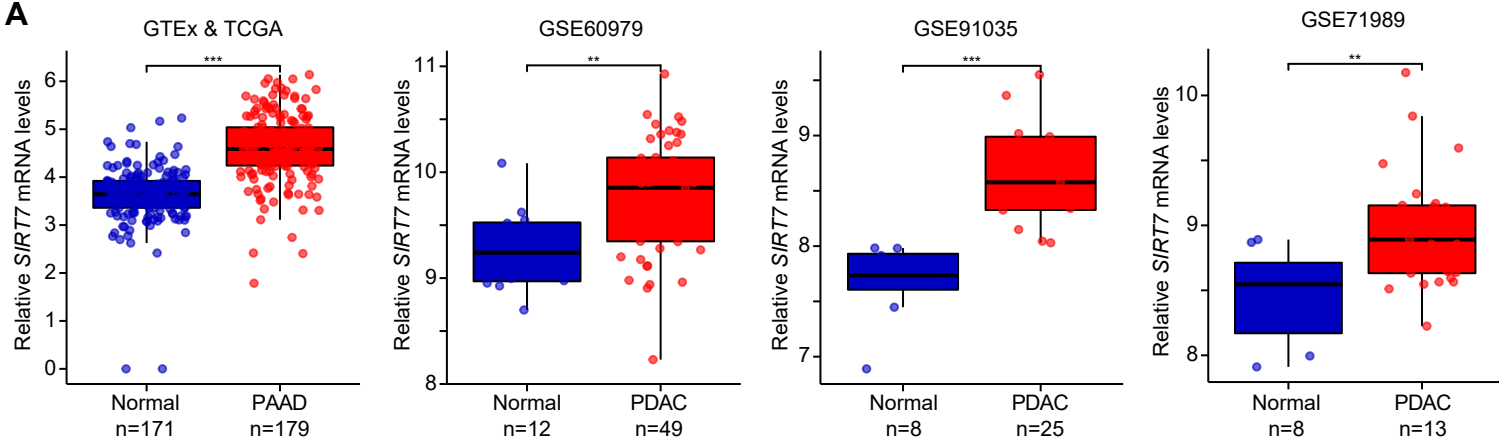**B**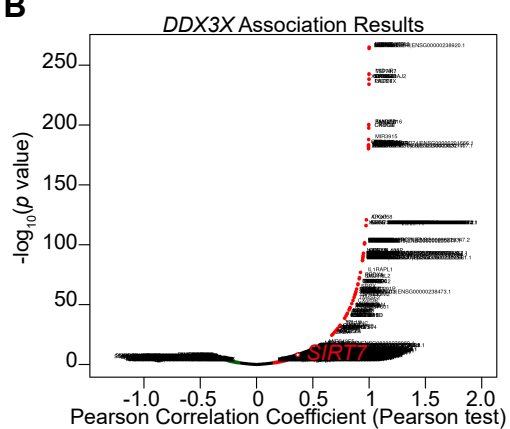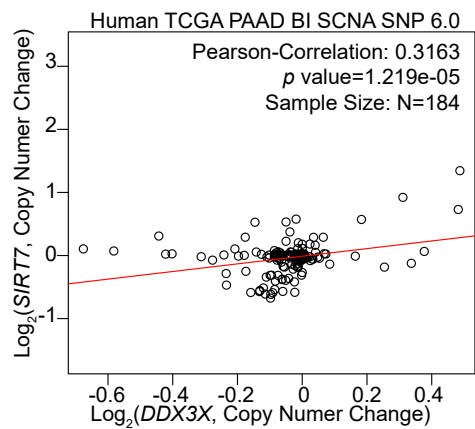**C**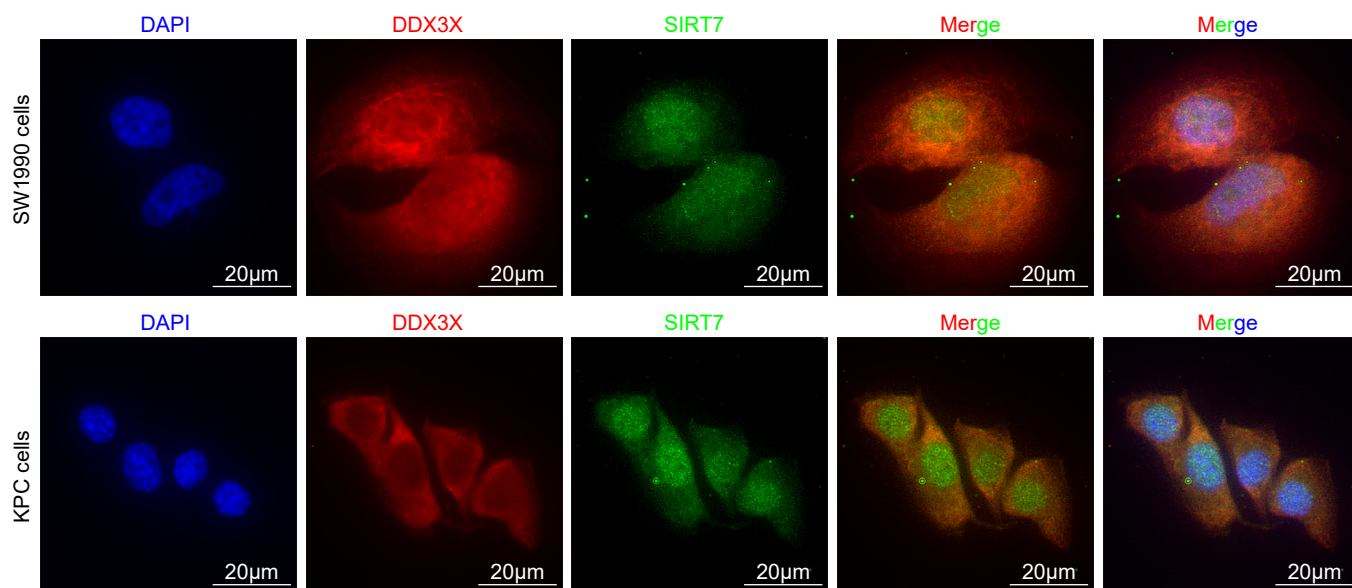

Supplement: Supplementary file 3 — Figure S2 [file 41389_2024_509_MOESM3_ESM.pdf]

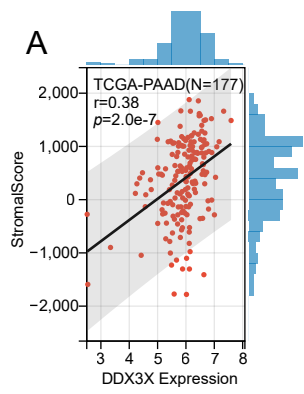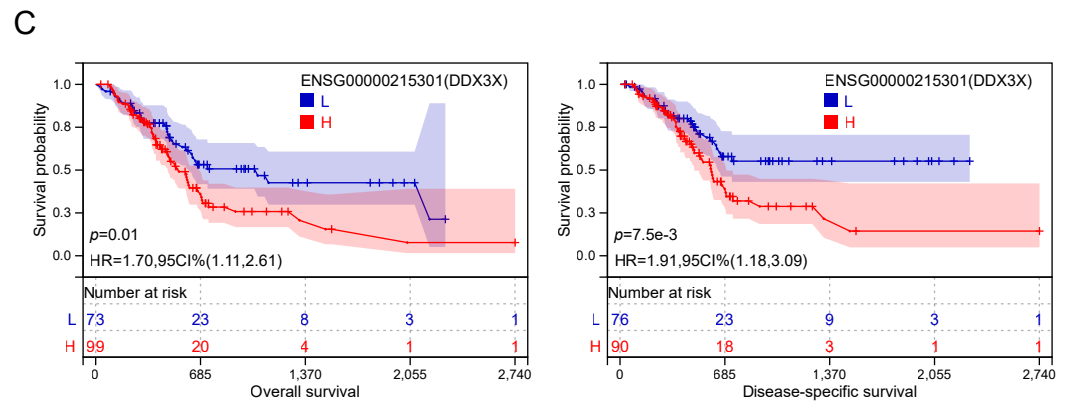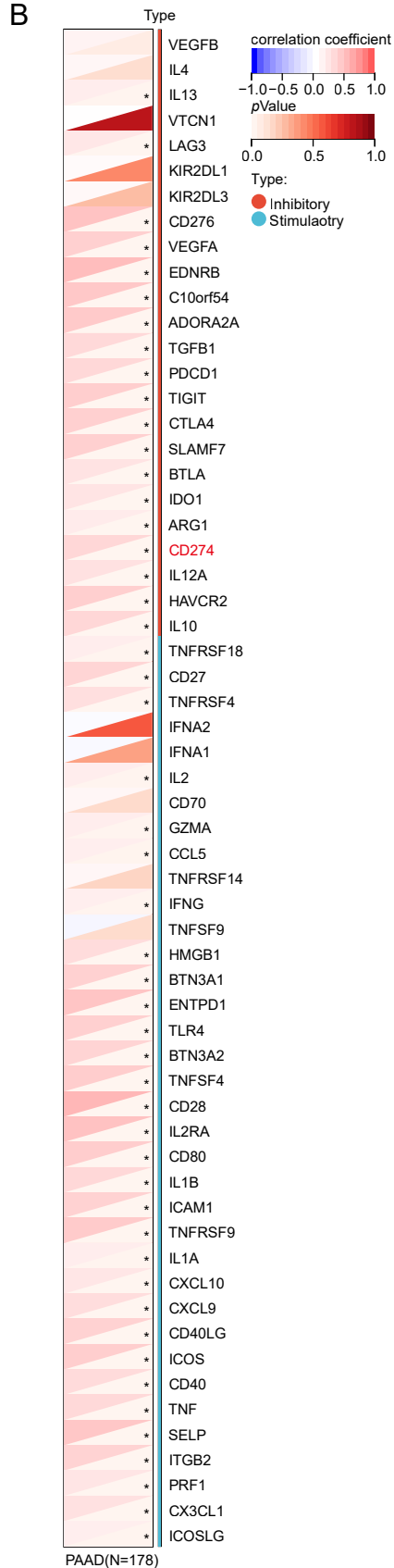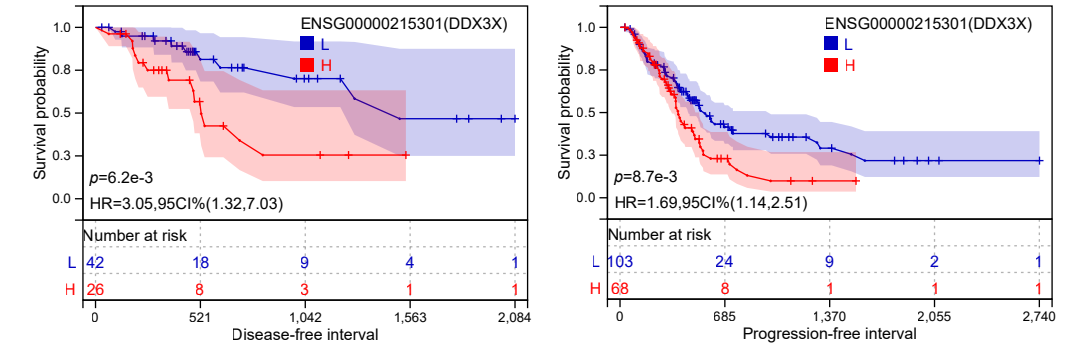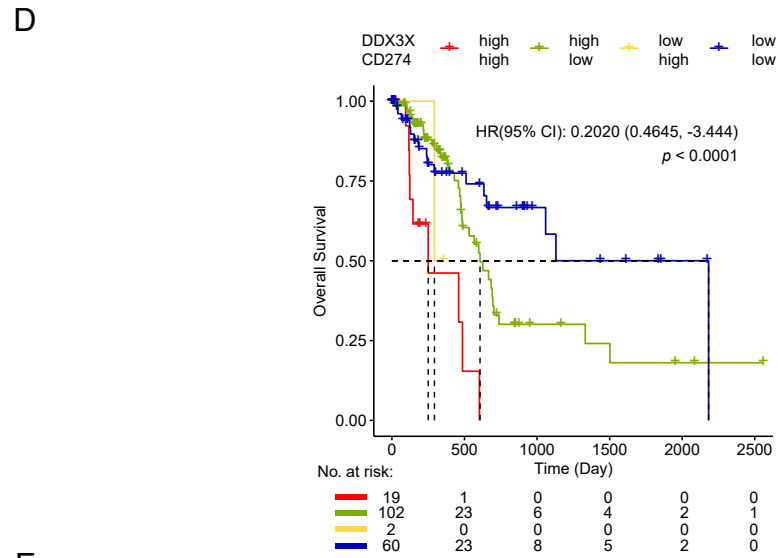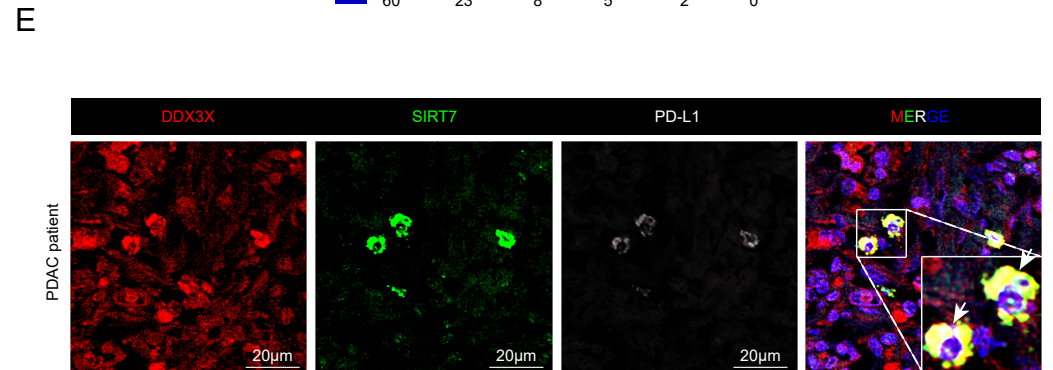

Supplement: Supplementary file 4 — Figure S3 [file 41389_2024_509_MOESM4_ESM.pdf]

**A**

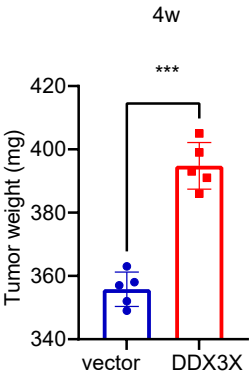

**B**

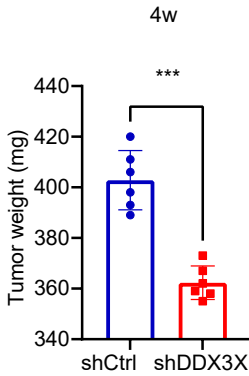

**C**

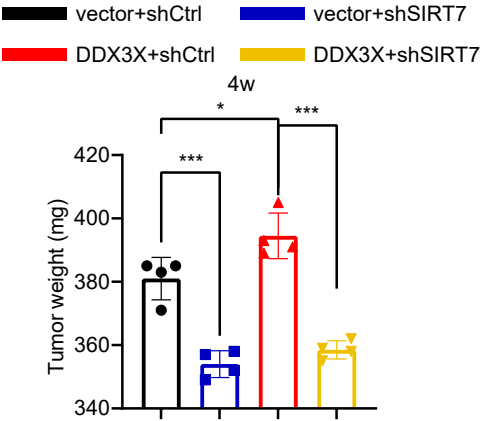

Supplement: Supplementary file 5 — Figure S4 [file 41389_2024_509_MOESM5_ESM.pdf]
