# Supplementary material for: DDX3X interacts with SIRT7 to promote PD-L1 expression to facilitate PDAC progression
Source: Oncogenesis. 2024 Feb 5;13(1):8. doi: 10.1038/s41389-024-00509-2 (PMC10844636; doi:10.1038/s41389-024-00509-2)
Supplement: Supplementary file 6 — Supplementary Table 1 [file 41389_2024_509_MOESM6_ESM.docx]

**Supplementary Table 1.** List of Primers for Real-time PCR

| Target | Gene ID | Primer | Sequence |
| --- | --- | --- | --- |
| *DDX3X* | 1654 | FP | 5’- ACTATGCCTCCAAAGGGTGTCC -3’ |
|  |  | RP | 5’- AGAGCCAACTCTTCCTACAGCC -3’ |
| *SIRT7* | 51547 | FP | 5’- TGGAGTGTGGACACTGCTTCAG -3’ |
|  |  | RP | 5’- CCGTCACAGTTCTGAGACACCA -3’ |
| *CD274* | 29126 | FP | 5’- TGCCGACTACAAGCGAATTACTG -3’ |
|  |  | RP | 5’- CTGCTTGTCCAGATGACTTCGG -3’ |
| *NEM1* | 58 | FP | 5’- AGGTCATCACCATCGGCAACGA -3’ |
|  |  | RP | 5’- GCTGTTGTAGGTGGTCTCGTGA -3’ |
| *COPS2* | 9318 | FP | 5’- CTGATGTGGAGAGCTTGCTGGT -3’ |
|  |  | RP | 5’- GGTTGGTCCATTTATCTAGTGCAG -3’ |
| *RPS20* | 6224 | FP | 5’- AGGACCAGTTCGAATGCCTACC -3’ |
|  |  | RP | 5’- CTCATCTGGAAACGATCCCACG -3’ |
| *RPS14* | 6208 | FP | 5’- GTGTCTGCCATATCTTTGCATCC -3’ |
|  |  | RP | 5’- GGTGAGGATTCATCTCGGTCTG -3’ |
| *RPS7* | 6201 | FP | 5’- GTTCAGTGGGAAGCATGTCGTC -3’ |
|  |  | RP | 5’- AGTCCTCAAGGATGGCATCGTG -3’ |
| *GAPDH* | 2597 | FP | 5’- GTCTCCTCTGACTTCAACAGCG -3’ |
|  |  | RP | 5’- ACCACCCTGTTGCTGTAGCCAA -3’ |

FP, Forward Primer; RP, Reverse Primer.
