# Supplementary material for: DDX3X interacts with SIRT7 to promote PD-L1 expression to facilitate PDAC progression
Source: Oncogenesis. 2024 Feb 5;13(1):8. doi: 10.1038/s41389-024-00509-2 (PMC10844636; doi:10.1038/s41389-024-00509-2)
Supplement: Supplementary file 7 — Details on other materials and methods [file 41389_2024_509_MOESM7_ESM.docx]

**Details on other materials and methods**

**Cell proliferation and colony formation assays**

Cell proliferation was assessed by a Cell Counting Kit-8 (CCK-8) kit (Vazyme, A311-01) according to the manufacturer’s instructions. Cells (1 × 10^3^ cells per well) were cultured in 96-well-plates for 0, 24, 48, 72, and 96 h, after which CCK-8 solution (10 μL) was added to each well, and the plates were further incubated for 2 h. The number of cells was quantified by measuring the absorbance at 450 nm on a microplate reader. Three independent experiments were performed.

For colony formation analysis, 1 × 10^3^ viable cells in different states were placed in 6-well plates and maintained in complete medium for 2 weeks. Colonies were fixed with methanol and stained with methylene blue.

**Cell invasion and migration**

For transwell invasion assays, 5x10^4^ cells were plated in the upper chamber (Transwell chambers, 8 mm pore size, BD Biosciences) in serum-free culture medium with Matrigel matrix (BD Biosciences) on the inserts. In the lower chamber, 10% FBS was used as the chemoattractant. After 24 hours, migratory cells on the lower membrane surface were stained with crystal violet and counted for three random fields per well.

For wound healing assays, cells were seeded in 6-well plates. Confluent cellmonolayers were scraped with 200 μL pipette tips and washed twice with PBS. Wound healing images were captured at 0, 24 and 48 hours after scratching. Cell motility was assessed by measuring the distance from each side of the cell wound. Images were documented under a phase contrast microscope.

**Stable knockdown and overexpression**

The shRNA and overexpression vectors for human DDX3X were synthesised by Corues Biotechnology (Nanjing, China). In accordance with the instructions of the product manual, Lipofectamine 2000 (Invitrogen, Inc.) was used to co-transfect the target plasmid or the scrambled vector, psPAX2 or PMG.2G, respectively, into HEK293T tool cells to obtain a DDX3X knockdown lentivirus or overexpression lentivirus. Then, the lentivirus (multiplicity of infection, MOI = 10) was used to infect PDAC cells. 48 hours after infection, the cells were further screened by treating them with puromycin (2 µg/ml, 72 h).

For knockdown of SIRT7 in PDAC cells, lentiviral plasmid vectors encoding short hairpin RNAs (shRNAs) targeting human SIRT7 and a negative control shRNA were purchased from Corues Biotechnology. Control (shCtrl) or DDX3X-knockdown (shSIRT7) cells were selected in Hygromycin B (500 ug/ml, 2w).

**Coimmunoprecipitation (Co-IP) assays**

The assays were performed as described previously. Cells were washed with cold PBS and lysed with cold cell lysis buffer for 30 min at 4 °C. Then, 500 μg of cellular extract was incubated with appropriate specific antibodies or normal rabbit/mouse immunoglobin G (IgG) at 4 °C overnight with constant rotation, followed by the addition of Protein A/G magnetic beads (Merck-Millipore, Darmstadt, Germany) and incubation for 2 h at 4 °C. Beads were then washed five times with cell lysis buffer (20mM Tris–HCl, pH 7.5, 150mM NaCl, 20mM KCl, 1.5mM MgCl2, 15% glycerol, 1mM EDTA, 0.5% NP-40, and 1% protease inhibitor). The immune complex was subjected to SDS-PAGE followed by immunoblotting with the indicated antibodies. Immunodetection was performed using Super ECL Detection Reagent (4A Biotech, Beijing, China) according to the manufacturer’s instructions.

**Docking**

To prepare the DDX3X and SIRT7 protein models for docking (PDB: 2I4I and 5IQZ, respectively), all solvent atoms and ligands were removed. Before docking, protein structures were optimized using Protein Preparation Wizard module (Small-Molecule Drug Discovery Suite 2019-1, Schrödinger LLC, New York, NY, USA 2019). Docking grids were centered to the C-terminal substrates. Ligands were prepared using OpenEye’s tautomers module, in order to assign the correct ionization and tautomeric form at pH 7.4 (QUACPAC 2.0.2.2. OpenEye Scientific Software, Santa Fe, NM, USA 2019).

**Immunohistochemical and fluorescent multiplexed immunohistochemical staining (MIF)**

The primary antibodies used for immunohistochemistry (IHC) and multiplex immunofluorescence staining (MIF) are presented in the online supplemental materials and methods.

For immunohistochemistry, paraffin-embedded and formalin-fixed tissues were used for immunohistochemistry detection using Immunohistochemistry Application Solutions Kit (ZSGB-BIO, SP-9001).

Fluorescent multiplex immunohistochemistry (mIHC) was performed using a PANO 5-plex IHC kit (Panovue, Beijing, China), according to manufacturer’s instructions. Different primary antibodies were sequentially applied, followed by horseradish peroxidase-conjugated secondary antibody incubation and tyramide signal amplification (TSA) using a TSA Fluorescence kit (Panovue, Beijing, China). After labeling with the human antigens, nuclei were stained with 4’, 6-Diamidino-2-phenylindole dihydrochloride (DAPI). Stained slides were scanned using the PerkinElmer Vectra 3.0 slide scanner (PerkinElmer, Shanghai, China), to obtain multispectral images. Fluorescence spectra were captured at 20 nm wavelength intervals from 420 to 720 nm, with identical exposure time.

**Quantitative real-time PCR analysis**

Total RNA was extracted by Total RNA Extraction Reagent (Takara, 9109) according to the manufacturer’s instructions. cDNA was synthesized by Reverse Transcription Kit (Vazyme, R323-01). qPCR was performed using ChamQ Universal SYBR qPCR Master Mix (Vazyme, Q711-02). The amount of each cDNA relative to the GAPDH endogenous control was determined using the 2^-ΔΔCt^ method. All primers used are illustrated in **Supplementary Table 1**.

**Western Blots and Antibodies**

Cells were washed in cold PBS and lysed in protein lysis buffer (4A Biotech, Beijing, China) for 0.5 h at 4 °C. The supernatant was collected after centrifuging for 10 min at 12 000 × g. The protein concentration was evaluated through the BCA protein assay kit. In brief, a total of 30 μg proteins were separated by 10% SDS-polyacrylamide gel electrophoresis and subsequently transferred to nitrocellulose membranes.

Primary antibodies used were mouse anti-DDX3X (sc-81247, Santa Cruz, 1:1000), mouse anti-SIRT7 (sc-365344, Santa Cruz, 1:1000), rabbit anti-DDX3X (ab235940, Abcam, 1:1000), rabbit anti-vimentin (ab92547, Abcam, 1:1000), rabbit anti-E-cadherin (ab76055, Abcam, 1:1000), rabbit anti-SIRT7 (29729-1-AP, Proteintech, 1:1000), mouse anti-GAPDH (AF0006, Beyotime, 1:10000) and rabbit anti-β-actin (AC026, Abclonal, 1:20000). Second antibodies were peroxidase-conjugated goat anti-rabbit and anti-mouse IgG (1:5000) (4A Biotech, Beijing, China). To visualize the proteomic bands, an enhanced western luminescent detection kit (4A Biotech, Beijing, China) was employed. The densitometry results were quantitatively analyzed by using the Image J software, with β-actin, GAPDH bands being normalization/internal controls as appropriate.

**Immunofluorescence**

Stable cell lines were seeded in 4-well Culture Slide (Millipore) to appropriate density. Paraformaldehyde were used to fix cells for 5min at room temperature and removed non-specific antigen by 5% BSA (Aladdin). Slides were incubated with primary antibody of DDX3X (ab235940, Abcam, 1:100) and SIRT7 (sc-365344, Santa Cruz, 1:50) at 4℃ overnight. Next day, cells were washed with PBS for three times, then incubated with secondary antibodies of CoraLite 488-conjugated Affinipure Goat Anti-Rabbit IgG (1:200, Proteintech, SA00013-2) and CL594-conjugated Mouse anti-rabbit IgG (1:200, Proteintech, CL594-66467) for 1h at room temperature. Cells nuclear was stained with DAPI (Yeasen, 40728ES10) for 10min at room temperature. Slides were coated with ProLong™ Gold and Diamond Antifade Mountants (Invitrogen, P10144). Images were scanned by FLUOVIEW FV3000 (Olympus).
